# “If you don’t behave, you’re in real shit, you don’t get outside the doors”—a phenomenological hermeneutic study of adolescents’ lived experiences of the socio-spatial environment of involuntary institutional care

**DOI:** 10.1080/17482631.2020.1726559

**Published:** 2020-02-12

**Authors:** Kajsa Nolbeck, Helle Wijk, Göran Lindahl, Sepideh Olausson

**Affiliations:** a Institute of Health and Care Sciences, Sahlgrenska Academy, University of Gothenburg, Gothenburg, Sweden; b Department for Quality Assurance, Sahlgrenska University Hospital, Gothenburg, Sweden; c Department of Architecture and Civil Engineering, Division of Building Design, Chalmers University of Technology, Gothenburg, Sweden; d Centre for Healthcare Architecture, Chalmers University of Technology, Gothenburg, Sweden; e Centre for Ethics, Law and Mental Health, Rågården Forensic Psychiatry Hospital, Sahlgrenska University Hospital, Gothenburg, Sweden

**Keywords:** Adolescents, institutional care, involuntary care, socio-spatial environment, photovoice, social exclusion, social control, phenomenological hermeneutics

## Abstract

In Sweden, according to law, adolescents with extensive psychosocial problems, substance abuse or criminal behaviour can be cared for in institutions. The two-fold aim of these institutions (to rehabilitate and incarcerate) puts special demands to their socio-spatial context.

**Purpose**: To elucidate adolescents’ lived experiences of the socio-spatial environment at special youth homes run by the Swedish National Board of Institutional Care (SiS) in Sweden.

**Methods**: Data collected through Photovoice and analysed employing a phenomenological hermeneutical method. Fourteen adolescents (age 15–19) were asked to photograph their environment, and this was followed up by in-depth interviews.

**Results**: Two themes emerged from the material: *the dense walls of institutional life* and *create and capture the caring space*. The socio-spatial environment can be seen as an additional “other” that distances the adolescents and the staff from one another. Negotiating with their behaviour, the adolescents strive to present themselves as worthy of increased degrees of freedom and ultimately access to the desired outside life.

**Conclusions**: In an institutional setting dominated by a security and criminal justice logic, words appear to have less impact than the environment. The adolescents appear to understand themselves through the socio-spatial other, causing reinforced feelings of social exclusion.

## Background

The question of how society handles “problematic youth”, as well as children and adolescents whose parents cannot or are unable to care for them, has been a recurrent issue in Sweden for decades (Hörngren, 2013). It is clear that the views and measures within social child and youth care in Sweden have shifted between social protection on the one hand and child welfare on the other (Hörngren, 2013; Leviner & Lundström, 2017). These two approaches become obvious in the socio-spatial context of special youth homes that encompass the aim of both rehabilitation and incarceration.

The Swedish National Board of Institutional Care (Statens Institutionsstyrelse, SiS), is responsible for special youth homes. It provides individual compulsory care for children and adolescents with psychosocial problems, substance abuse and/or criminal behaviour, and enforces punishment in accordance with the law on young offenders (SFS 1998:603, 1998; Swedish Government Offices Social Affairs, 1990). There are 23 youth homes in Sweden today, with about 700 places (The Swedish National Board of Institutional Care (Statens Institutionsstyrelse S), 2017). A particular characteristic of the special youth homes, compared to other care institutions, is the staff’s right to exercise power to, for example, avert threats and violence (see §15, Law on the care of the young people) (Swedish Government Offices Social Affairs, 1990). In the context of youth institution incarceration, this could be seen as an expression of power that is intertwined with rehabilitation and care, and which constitutes two contradictory logics (Silow Kallenberg, 2016). The youth institution is similar to what Goffman describes as the total institution—a social hybrid separated from the wider social context of the community and a world of its own where work and residence take place behind the same walls (Goffman, 1991). Foucault designates power as dependent on the environment, since the ordering of spaces, physical objects and people within those spaces, facilitates certain social roles, actions and interactions, while obstructing others (Foucault, 1982).

The socio-spatial environment in this study is defined as the intertwined objective and social dimensions of space, where space is not merely objective and physical but also social in the sense that human action is always embedded in spatiality. This means that the objective, physical and spatial space is intertwined with the subjective and social space, and together they constitute and determine social actions (see, for example Schatzki, 1991; Zieleniec, 2007) (Schatzki, 1991; Zieleniec, 2008). Moreover, place and space are fundamental perquisites for human existence from a lifeworld perspective. Lifeworld is the individual’s embodied perceptions and experiences of everyday life. Hence, spatial and physical spaces are intertwined with and inseparable from the body; the body inhabits space, place and time and shape individuals’ experiences of the world (Bengtsson, 2001).

Previous research on the impact of spatial environments on rehabilitation and wellbeing have shown stress reducing effects associated with design solutions, such as decreasing crowding and noise, promoting private areas and facilitating staff observation in healthcare settings (Ulrich, Berry, Quan, & Parish, 2010; Ulrich et al., 1991, 2008). Previous studies have also shown that environmental factors relating to healthcare unit atmosphere co-create a sense of belonging (Borge & Fagermoen, 2008; Evans, 2003). Furthermore, the existing body of knowledge deals almost exclusively with the effects of built environment factors on individuals’ health outcomes in healthcare settings, including psychiatry and forensic psychiatry. In the field of social work, the focus is mainly on the prison environment (Huey & McNulty, 2005; Inderbitzin, 2007; Moran, 2013a, 2013b), and research concerning the general interplay between social and spatial factors and the specific impact on children and adolescents is sparsely explored. The existing body of knowledge in the context of the involuntary institutional care of children and adolescents focuses mainly on security, risk factors and individual outcomes of care interventions (Huey & McNulty, 2005). However, there are some research regarding the importance of the physical environment as a symbolic expression of culture and philosophy in the institutional care of children (Bailey, 2002). All in all, this clarifies the lack of research on young people’s lived experiences of the socio-spatial aspects of compulsory care.

To the best of our knowledge, there are no current studies exploring the impact of the socio-spatial environment on institutional involuntary care for children and adolescents in Sweden. The aim with the study is, therefore, to elucidate the adolescents’ lived experiences of the socio-spatial environment at special youth homes run by SiS in Sweden. In the current paper, the terms physical environment and spatial environment refer to the objective, material environment, while the term socio-spatial environment designates the intertwining of physical, spatial and social dimensions of space. The study is part of a research project that examines the impact of the physical environment on the rehabilitation and wellbeing of adolescents under institutional care.

## Methodology and method

A qualitative explorative method was chosen for this study. Data was collected through Photovoice (Novek, Morris-Oswald, & Menec, 2012; Walton, Schleien, Brake, Trovato, & Oakes, 2012; Wang & Burris, 1997) and analysed by employing a phenomenological hermeneutical method (Lindseth & Norberg, 2004). Photovoice is an actor-oriented co-participatory research methodology that is particularly suitable for research with vulnerable groups, such as adolescents under institutional care. Moreover, the focus of the study further justifies the choice of method, since it enables the respondents to put words to their experiences (Sitvast, Abma, Widdershoven, Lendemeijer, & Stories, 2008). The method has also been shown to support young people in expressing their feelings about abstract phenomena such as the socio-spatial environment. Respondents are first given cameras to document various aspects of their environment in relation to the research questions; this is followed up by in-depth interviews (Kvale, 2009) that use the photos as the starting point (Novek et al., 2012; Walton et al., 2012; Wang & Burris, 1997).

Purposeful sampling was employed (Polit, 2016) as follows. The head office of the institutional youth homes (SiS) consented to the project and informed the special residential homes that they would be contacted by the research team. Prior to the study, a number of pilot interviews were conducted to test the method in the adolescent institutional setting (James & Olausson, 2018). The pilot study aimed to evaluate the process of data collection in special residential youth homes, as well as if Photovoice would be suitable for the targeted group. The pilot study resulted in some minor method adjustments, such as; being responsive to the youth’s need to take breaks and adjustment of the information letter. The pilot interviews are not included in the current paper, but published elsewhere (see James and Olausson, 2018) (James & Olausson, 2018). Thereafter, homes were selected for data collection according to various geographic location and with different types of physical and environmental characteristics, such as the degree of lock-in and urban or rural location. Ten homes were included in the larger research project. Due to the different institutions’ needs and opportunities at the time of data collection, it was decided that data collection for this study should be conducted at eight different units within two different residential homes. The data collection procedure begun by with informing the heads of the residential homes about the present research project, the aim and the data collection methods, and ethical approval was requested. At the same time, the managers at each unit were informed and agreed to introduce the researchers to the staff at each unit.

The researchers spent an extensive time at each unit to bond and build trustful relations with the children/adolescents. This was done by first introducing themselves and the project and stressing its voluntary nature for all the children and adolescents. The researchers then spent time with the children and adolescents—playing games, talking or just sitting in the social areas, for example. Several children/adolescents showed interest in the research project, and, consequently, the researchers informed them orally and in writing once more about the aim of the study and its voluntary nature. The children and adolescents were given the opportunity to ask questions and time to consider the invitation.

In total, 14 children and adolescents (six boys and eight girls) aged 15–19 years consented to participate in the study. Any of the children who wanted to participate were welcome to do so. Children and adolescents who agreed to participate were given a polaroid camera and were asked to take photos of various aspects of their immediate surrounding that they associated with a feeling, regardless of whether it was positive or negative. The researchers were present while photos were taken, and the participants were informed that no photos were allowed of other people at the unit.

The follow up in-depth interviews mostly took place in the respondents’ bed rooms (except for one adolescent who wanted to sit in the conversation room) and used the photos as the starting point. The interview started with open-ended introductory questions, where the adolescents described the pictures and why they took them. The interviewer followed up by regularly asking for stories and examples, noting keywords, reoccurring themes and emotional reactions. The researcher’s position constituted of an open “not knowing position”, that attempted to reveal the meaning of the phenomenon (the socio-spatial environment) to the individual (Dahlberg, Dahlberg, & Nyström Pettersson, 2008). Three of the adolescents did not take photos, in those cases the interviews started with an open-ended question were they were invited to share their experience of their living space and bed room. For practical and ethical reasons, two researchers were generally present during the interviews. As part of the relationship building process, and as a power equalizing strategy, the researchers asked the adolescents where to sit—i.e. the respondent could place the researcher at whatever distance or in whatever place they desired. This mostly resulted in the researchers sitting on the floor and the adolescent on the bed or a chair. In general, one of the researcher led the interview, while the other asked shorter supplementary questions. The interviews took approximately one hour and were recorded and transcribed verbatim. Seventy-eight photos were taken by 11 respondents (five boys and six girls), while three adolescents refrained from taking photos. In all cases, the adolescents were allowed to keep their photos, and the majority chose to do so. In those cases, the researchers photographed the adolescents’ images for the documentation. The data was collected between 28th of February and 11th of June 2018.

A phenomenological hermeneutical (Lindseth & Norberg, 2004) method based on the interpretation theory of Ricœur was chosen to analyse the data. This method strives to interpret the text in a logical systematic and structural way (Ricœur, 1976). The interpreter seeks to reveal the meaning of the text through the interpretation process, which implicates the dialectic movement between the whole and the parts in the hermeneutic circle (Dahlberg et al., 2008). According to Ricœur, to create valid knowledge of lived experiences and life-world necessitates both understanding and explaining the text.

The analytical process consisted of three methodological steps. First, the texts (i.e. the transcribed interviews) were read through several times with an open mind and an empathic approach. This phase is called the naïve reading and seeks to grasp the text as a whole and understand what the text is about so as to formulate a preliminary interpretation, or a qualified guess, which will be the subject of further scrutiny. According to Lindseth and Norberg, a narrative, empathic style of writing can be employed to convey to the reader a sense of what the phenomenon is about (Lindseth & Norberg, 2004).

**Figure 1.**
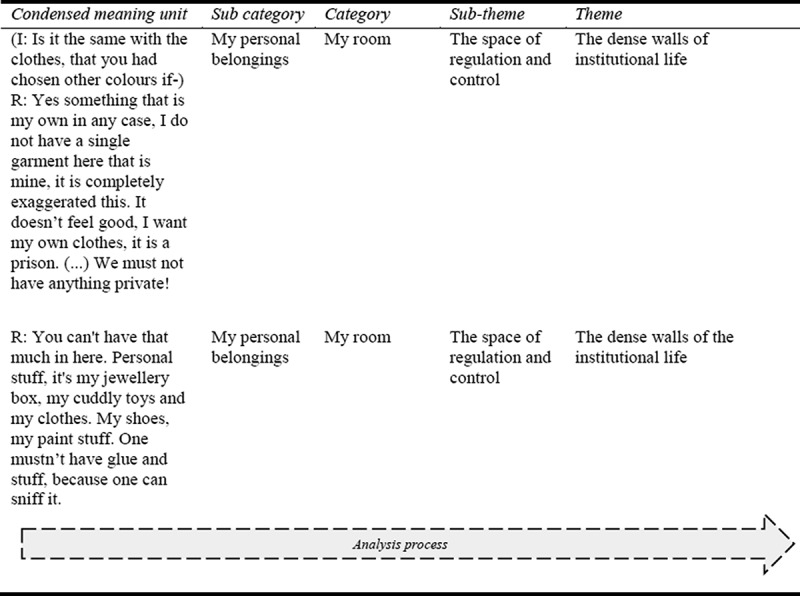
Overview of the analysis process. The figure shows an overview of the process from condensed meaning unit to theme, from the first and second structural analysis, according to the phenomenological hermeneutic method of Lindseth and Norberg

Second, structural analyses were conducted to examine and explain the naïve understanding. This phase is characterized by a critical approach to validate or falsify the naïve understanding. The structural analysis was based on a systematic reading, whereby meaning units were identified, condensed and critically reflected upon to allow the meanings of the text to emerge and to categorize and abstract them into subthemes and themes. The process of the structural analyses, according to the phenomenological hermeneutic method of Lindseth and Norberg (Lindseth & Norberg, 2004), is shown in Figure 1.

Lindseth and Norberg emphasize that several structural analyses may be performed during the interpretation process as the text always has a surplus of meaning (Lindseth & Norberg, 2004; Ricœur, 1976). The structural analyses can be conducted thematically or categorically depending on the character of the text and the research phenomenon.

Third, the whole text was read through again with a critical approach and both the naïve reading and the themes in mind. The text and the analysis were reflected upon in relation to the research question and relevant literature, and, finally, a comprehensive understanding of the phenomenon of interest, was reached (Lindseth & Norberg, 2004). In the current study, three structural analyses were performed. The first was performed on the photos and is not presented in this paper, instead the photos are presented as part of the second structural analysis. The analysis process was mainly carried out by the first and last authors, in that the entire text material was read by both, the first author formulated the naïve understanding and conducted the structural analyses with close supervision and in collaboration with the last author. In connection with each analysis step, the results were presented, discussed and revised together with the second and third authors. The naïve understanding and the structural analyses two and three are presented in the results section below.

## Ethical considerations

Although the project was approved by the Ethical Review Board (ID nr 1158–16, 2017-03-06), there was an ongoing dialogue within the research group regarding ethical questions throughout the research period. Due to ethical considerations and child rights perspectives, research in the context of involuntary institutional youth care puts high demands on researchers (Källström, 2017). The adolescents were invited to participate in the study through oral introduction by the researchers and written information about the project. Their right to withdraw at any point without reason and without consequences for their care was stressed. The target group involved in the research are persons in a vulnerable position; therefore, an analysis of the ethical risks was conducted to identify and prevent possible ethical dilemmas in advance. Through dialogue within the research group an ethical codex, was discussed and decided upon. The ethical codex included a step-by-step action plan for how the researchers should act if they observed or received information about harm, neglect or abuse. The foundation of the ethical codex constitutes of principles on research ethics including respect for autonomy, non-maleficence, beneficence and justice (Beauchamp, 2013) as well as a child rights perspective (United Nations Human rights office of the high commissioner, 1989). Informed consent was collected at the time of data collection.

## Findings

### Naïve understanding—life on hold

The following presents the naïve understanding of the interview texts and photos construed as a narrative. This constitutes the first step of the analysis process (Lindseth & Norberg, 2004).

The institution’s world is a life on hold. “In here” it is all about “doing my time” so that I can get out of here and “have my own” life and place. My life is out there, in “the reality”, a place that I long for. Time seems strange in here, ungraspable. My narrative and chronology is messy and incomplete; I want to feel at home, but everything is just temporary. I have learned from experience to never anchor in a place for a long time. It feels like a vacuum, waiting for life to start again. In here, I become someone else. The knowledge of what I miss is tangible; it feels like losing parts of my identity.

“In here”, the spatial environment is usually dirty, terrifying, cold, hard, uncomfortable, outdated, filled with odours and compromises, a forced collective engagement, where adaptation, rules and routines seem to be superior. The institution’s spatial environment hurts, scatters, limits and punishes; it describes what I am and who I am. But who I really am as a person is subordinate, invisible or forgotten. Everyday spaces become a commodity and something I must deserve; I bargain with my behaviour, but whether I get access or not seem dependent on the staff’s unpredictable goodwill.

Everything I say and do is observed and evaluated. But mostly, I do nothing; the institutional environment is characterized by boredom and lack of activity. A passivating environment that limits my ability to move, both physically and mentally, both literally and metaphorically. Boredom creates hopelessness, restlessness, frustration and anxiety. I feel powerless when I am not listened to about my needs.

Sometimes, however, I find myself in a close and trusting space. When someone in the staff does a little extra, sees me for who I am, gives me warmth and consideration, I feel embraced. It gives hope. Or sometimes I create my own familiar space together with one of my peers. However, mostly the staff’s power creates a relational distance between them and me that undermines and complicates trusting relationships. Nevertheless, I try to resist; I make doodles on my walls and my bed, cover my walls with pictures and quotes and use the poor interior design in new creative ways, re-naming the ugly, despicable environment. Using the spatial environment in my own way becomes a way to take control and establish ownership over my room. Pictures and personal belongings (when I am allowed to have them) make me (re)take power over my room; they are my memories and reminders, give thoughts of past and future, but also illustrate my desire—a link to life out there that enables a mental movement. They give rise to anecdotes that fulfil a function of the story of myself as a person—an attempt to fill in the gaps in my narrative, which often lacks witnesses. They can tell a story of who I am, who I was, who I could be and who I want to be.

### Structural analysis II—a description of the socio-spatial environment

To further examine the naïve understanding, i.e. to validate or falsify the preliminary interpretation, structural analyses according to the method were performed. This step is characterized by an objective/structural or critical attitude to explain the naïve understanding (Lindseth & Norberg, 2004). The first structural analysis was characterized by the sorting and categorization of photos to explicate *what* the text and photos express about the phenomenon of interest (Ricœur, 1976). This structural analysis is not presented in the paper; instead, the photos are displayed in the second structural analysis. The text was then gone through, and units of meaning were identified and condensed, i.e. they were shortened as much as possible without losing their meaning. The condensed meaning units were then sorted and categorized according to similarities and differences, together with the photos, resulting in three main categories with several subcategories (Table I). According to Lindseth and Norberg the material was considered as strictly as possible, with the authors adopting a critical attitude and striving to control their preunderstanding (Lindseth & Norberg, 2004).

**Table I.** Overview of subcategories and categories—Structural analysis II

Number of photos	Subcategory	Category
12	My personal belongings	My room
9	Décor and walls	
6	The bed	
2	Sound, light, air and temperature	
8	Furniture	
8	The window and the view	
17	The sanitary spaces	Within the institution
8	The kitchen, living room and other common spaces	
-	The rooms of the other unit inhabitants	
-	The school and the treatment spaces	
-	The gym and the sports area	
4	Other spaces	
4	Outdoor and patio	Outside and beyond the institution
-	Outside the institution area	
-	Beyond the institution placement	

The table shows the categories and subcategories found in the second structural analysis according to the manifest content of the data material. The table also shows the number of photos taken by the informants for each category. For security and ethical reasons no photos were taken for some categories, such as the rooms of the other unit inhabitants.

#### My room

The rooms are private; however, the bathrooms are often shared. The colours of the room (the colour of the walls, the curtains or the blind) are often described as dull, boring, hospital-like and depressing. The adolescents expressed a wish to decorate their rooms; re-order the furniture, “put something” on the walls and have their personal belongings; and have their own clothes, photos, posters or a stuffed animal from at home—in summary, a room decorated by themselves and to their own taste. They want more “fun” and more “life” in the room. When personal belongings are allowed, they have a prominent place in the room. This also includes things that the adolescents have created themselves or, in some cases, received from an important staff member. However, the opportunity to decorate differs between units and institutions, and while some of the adolescents have covered their walls with pictures and quotes (mostly girls), others have put their own tag on the wall (mostly boys).

**Photo 1.**
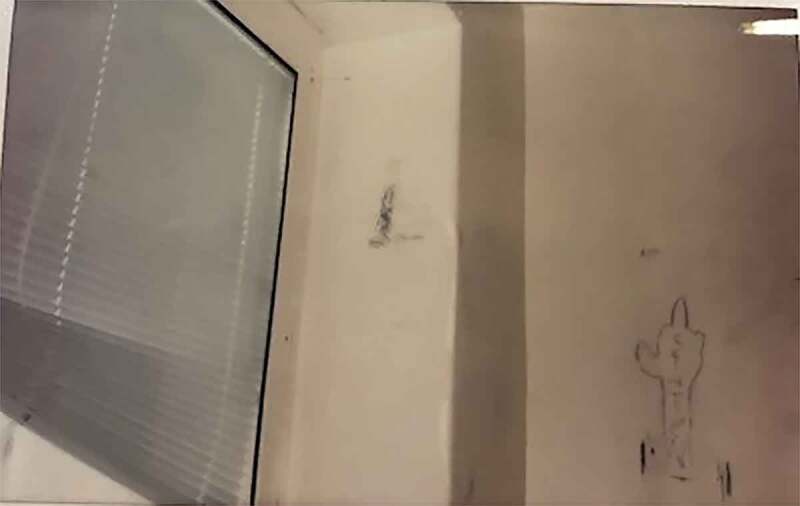
Photo taken by a young male depicting a window with a stuck blinds and doodles on the wall

**Photo 2.**
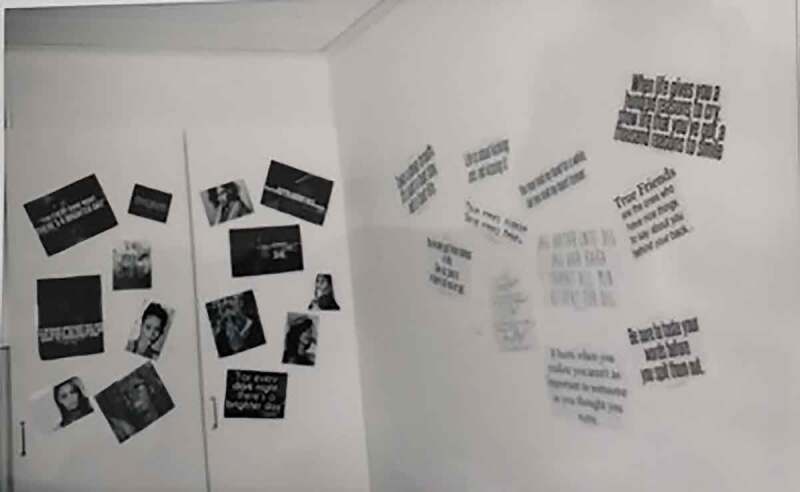
Photo taken by a young female showing a wall decorated with photos and quotes

**Photo 3.**
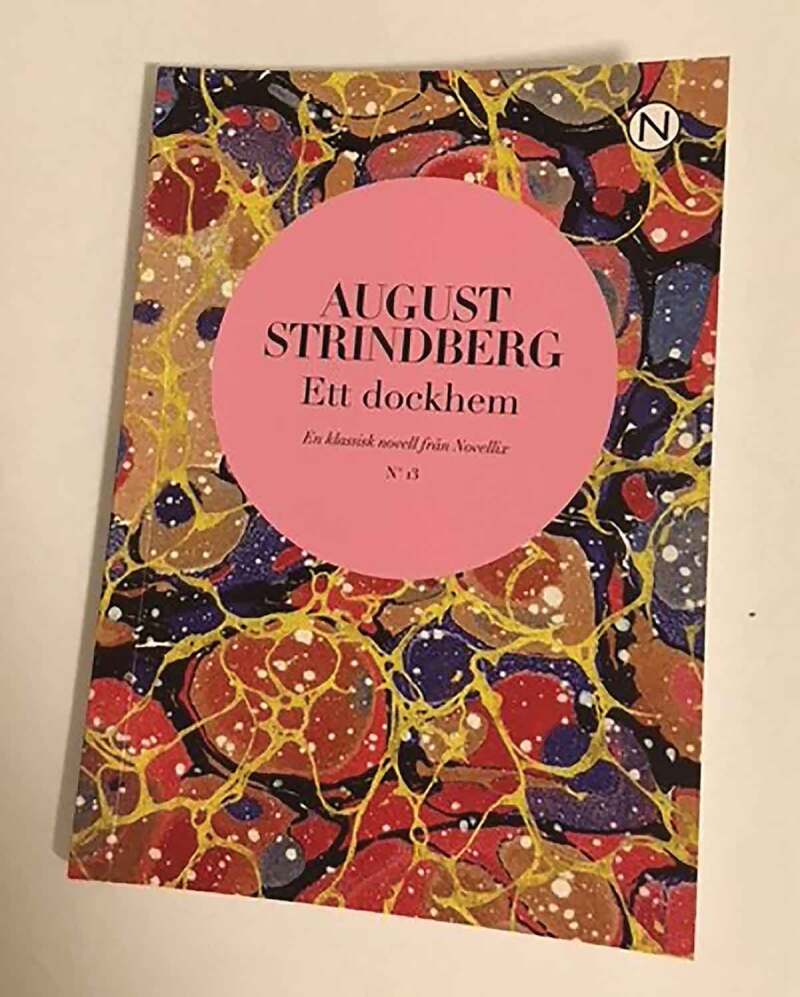
Photo taken by a young female, displaying Strindberg’s short story “A dollhouse”

The furniture in the room often consists of a bed, sometimes a bedtime table, a desk with a chair, sometimes an armchair and often an open wardrobe or shelves. The furniture is often fixed to the wall for security reasons. The most talked about piece of furniture is the bed and the mattress. The fireretardant mattress was described as tough, noisy and uncomfortable, causing back pain, poor sleep and sometimes nightmares, despite the extensive use of sleeping pills. The bed is too small—smaller than their referent bed (the bed at home or the bed wished for)—and used in many ways: for writing, listening to music and watching TV, which is allowed at some units. Some adolescents describe smuggling in food and eating in their bed at night.

The furniture is often broken, not working properly and not what the adolescents would have chosen themselves. This also reflects in the adolescents’ stories about using the furniture for other purposes, such as turning an armchair into a bed when they sneak in to each other’s rooms at night.

**Photo 4.**
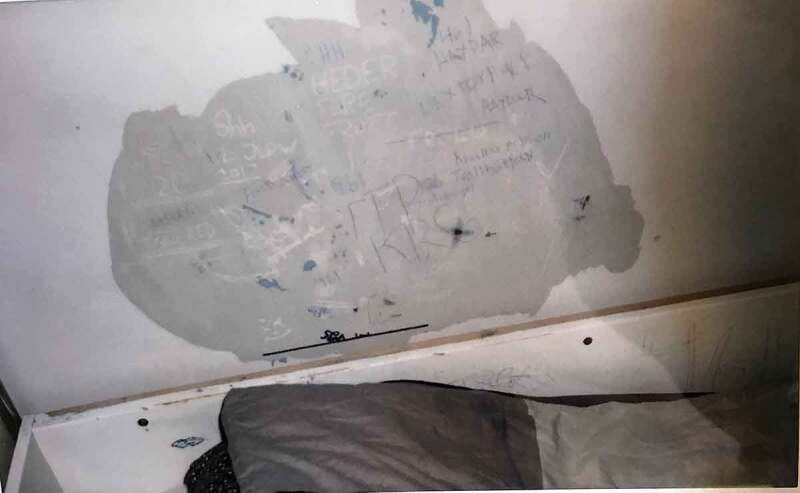
Photo taken by a young male showing the bed and a partially destroyed wall with doodles on it

**Photo 5.**
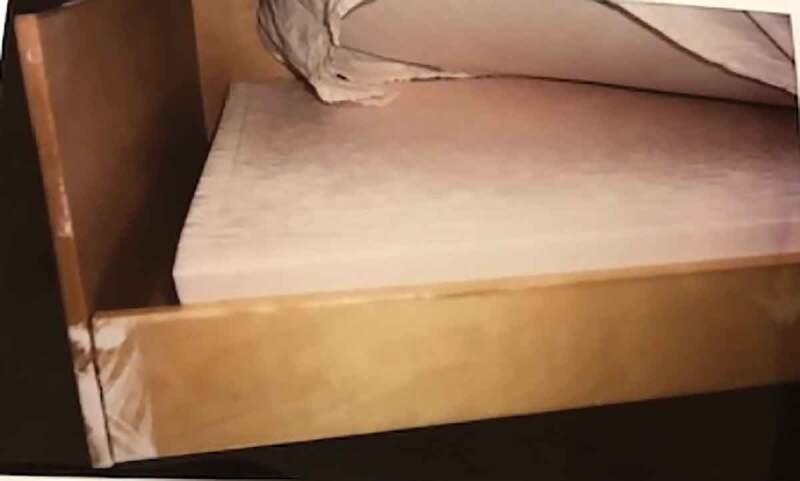
Photo taken by a young male showing the bed with a fireretardant mattress

Regarding the regulation of air and heat, radiators and ventilation are often stuck in a certain position, not working properly and out of one’s control. The bad air or lack of air and temperatures that are too hot or too cold cause headaches, heavy breathing and itchy eyes and nose. All rooms have at least one window and sometimes have blinds or curtains, which are often (if they are not broken) pulled down, creating a dark or semi-dark room. The adolescents do not want anyone passing by to see in or do not themselves want to see out to the fence and barbed wire. Some say it is just more cosy that way.

#### Within the institution

The sanitary spaces (i.e. bathrooms, toilets and showers) are often described as boring, ugly and not working properly; more often, they are described as disgusting, despicable and full of graffiti. Wishes to “do something about” the sanitary spaces range from decoration (adding more colours, plants or candles) to cleaning up and renovation. Opinions about other spaces, such as the living room and the kitchen, differ, but they are often expressed as dull, boring (living room) or dirty and not properly cleaned (kitchen). While the living room is often an open space to which the adolescents have access during the hours that they are not locked up in their rooms, the kitchen is often only accessible to the adolescents during specific hours.

**Photo 6.**
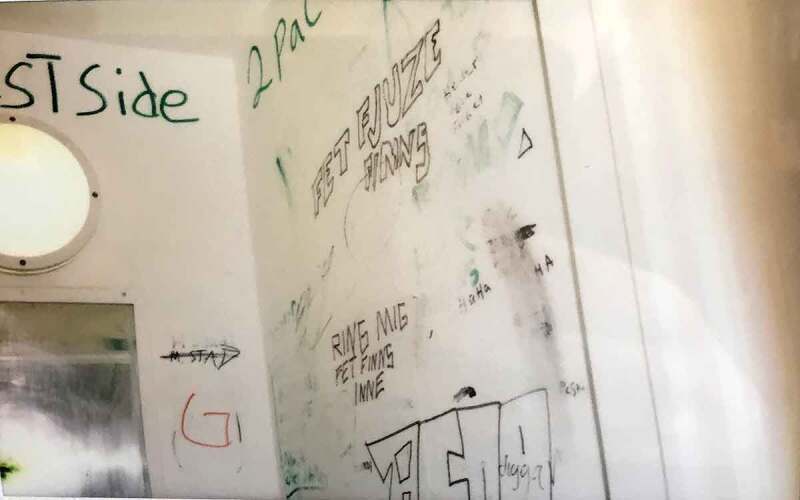
Photo taken by a young male showing a bathroom with doodles on the walls

**Photo 7.**
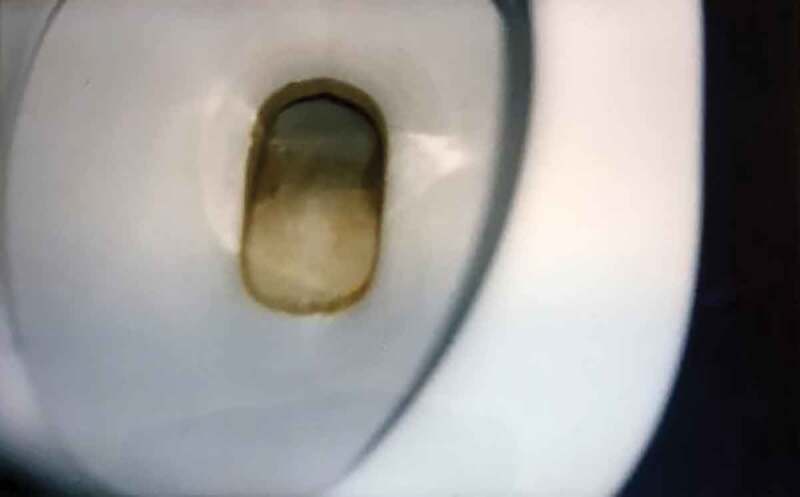
Photo taken by a young female showing a dirty toilet

Other rooms talked about in some interviews were the space for seclusion and the space for individual care. These spaces are similar to the extent that they separate the adolescent from the other adolescents at the unit for a specific length of time. While the space for seclusion is described as containing almost nothing, besides a mattress on the floor, the space for individual care does contain things, such as a TV and a sanitary space (often without a door). Other spaces mentioned are the gym or the sports area and the school. Some adolescents express that they only have access to the gym or sports area at specific hours, while access is more freely organized at other units.

#### Outside and beyond the institution

The opportunity to get outside the doors of the unit is frequently mentioned in the interviews—whether to access the fenced patio or terrace, the smoking area or the rest of the institutional area (taking walks, etc.) or to go for a car ride, go home occasionally or leave permanently.

**Photo 8.**
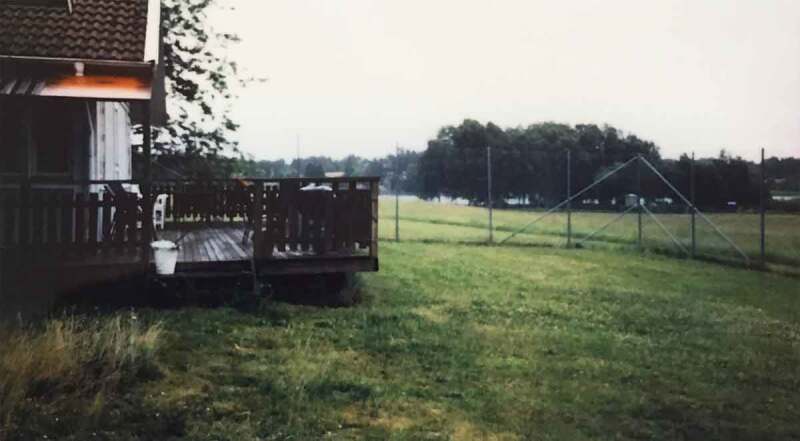
Photo taken by a young female showing the department’s fenced back with patio and lawn

### Structural analysis III—the meaning of socio-spatial aspects of institutional everyday life

After the first and second structural analyses the next step, following Ricœur’s theory of interpretation, was to explain and unfold the meanings ascribed to the phenomenon of interest (Ricœur, 1976).

Two themes (Table II) emerged from the interviews, both of which involved spatial and concrete dimensions and abstract and existential dimensions. The themes and subthemes can be directly linked to a specific spatial location or arise in a particular context depending on spatial and social circumstances, attributes and symbols. Thus, different themes and subthemes may appear in the same spatial locations, or certain themes and subthemes may be more prominent in particular locations.

**Table II.** Overview of subthemes and themes—Structural analysis III

Subthemes	Theme
The space of regulation and control	The dense walls of institutional life
The space of alienation and powerlessness	
The space of scarceness and passivity	
The space of loneliness and seclusion	
The space of trust and familiarity	Create and capture the caring space
The space of hope, longing and desire	

The table shows the themes and subthemes found in the third structural analysis according to the latent content of the data.

#### The dense walls of institutional life

The residential youth home presents itself to the children and adolescents as a *space of regulation and control*, evoking feelings of frustration and loss of control. Places where rules and routines emerge as linked to space and time were often perceived as difficult to understand and unpredictable and were associated with a clear and intrusive awareness of the power imbalance between the staff and the adolescents, where the latter were constantly inferior. However, under certain conditions, the rules sometimes create a sense of safety. The stories reveal that the children and adolescents are controlled both in space and time. This manifests itself as limiting liberty of action, such as being unable to move freely from inside to outside the unit and limited opportunity to control various areas of space and its characteristics. As one of the adolescents expresses it: “*The longest you can move inside the department is maybe 50 meters (…); you panic that you are so trapped. But you can’t do anything about it and that just makes it feel even worse*.” The environment is adapted to the security ideal rather than to fit the needs of the adolescents. This evokes feelings of anxiety and powerlessness which are associated with being trapped in a fusty space. The adolescents negotiate with their behaviour and identity to try to escape the regulated and controlling environment of the institution, constantly aware of their inferiority.

If you don’t behave, you’re in real shit, you don’t get outside the doors.

Actually, it is only required that the staff is in a bad mood that day, so they put you there (in seclusion) for the smallest thing. If you drop a potato, they put you there. (…) You know, in order to put a youth in individual care, a lot of paper is required. (…) But for seclusion, nothing is required …

The residential youth home further materialized itself as a *space of alienation and powerlessness*, which gave birth to feelings of hopelessness, frustration, powerlessness and sometimes aggression. These feelings were manifested through stories about disgusting hygiene facilities, being distanced from interior aspects such as colour choices, or perceiving furniture as not functional or appealing. In addition, they manifested in terms of alienation from society, social conventions and the possibilities of a normal life. Alienation is almost always closely linked to an expressed sense of powerlessness, both spatially (there is no point in trying to influence the physical environment) and existentially (have tried everything but still did not succeed). The alienating environment changes the person, making her/him un-recognizable in her/his own eyes and thus also creating feelings of alienation in relation to the self.

But I advise not to end up here at all, not because it is not good here but you get stupid in your head. The environment, and the doors are locked and all strange routines and that they lock in the evenings (…) Four years ago when I ended up on my first placement I was not the one I am now (…) You are not as happy and you are not same person at all.

The adolescents also reflected on the institutional space as a *space of scarceness and passivity*. Both the spatial environment and the content of the day to day are materialized as a dull, depressing and passive existence; an existence characterized by waiting and inactivity and being dejected. Moreover, the residential home appears to be a *space of loneliness and seclusion*, with a shifting character that depends on various situations (spatial as well as social). The residential home is a vessel for contradictory feelings and grief, loneliness and/or abandonment. However, it is also a space where the children and adolescents can feel safe and have privacy.

The (bed) is uncomfortable. It’s hard. (…) The (body) feels stiff, I have pain. I dream every day that I am out and when I wake up and just ‘fuck, I’m left in hell’. It’s depressing, you just go here …

For example, the private bedroom shows itself to be both a safe space to be left alone and a depressing and anxious space where one is locked in with one’s thoughts and feelings and left behind. Whether the residential home or the private bedroom become spaces of seclusion or spaces of loneliness is regulated in space and time and is almost never a personal choice. Pulling down the blind; wanting to close in, foreclose and create their own space; and using spatial and physical attributes are sometimes ways to create a space to reflect and contemplate. Loneliness often becomes intrusive and desperate.

The most difficult moments are when you have to go to bed in your room. Really. All thoughts come, you get anxious, you get stressed, you feel that you have not so fucking many. You feel very powerless. Being able to call someone when you feel bad, you do not have that opportunity. (…) It is not on the map, but you know that you need to lie inside the room until it is morning and you have no alternative. I know some, they have made chaos so the staff have had to take them out. It needs to go that far.

#### Create and capture the caring space

However, sometimes there is a glimpse of trust, hope and desire that cracks the dense walls of the institution. The residential youth home can also present itself as an *embracing space of trust and familiarity*. The stories reveal a desire and a quest for possible close relations and moments of trust. Although feelings of trust and familiarity are most often distant and out of the children and adolescents’ reach, they sometimes materializes in the day-to-day life of the institution. The existence of close and trusting relationships with other young people at the institution or with staff creates feelings of recognition, warmth and a sense of belonging. However, while these relations and moments are generally absent, the adolescents are left to create these enclosing spaces for themselves. An embracing sense of secrecy then appears through physical attributes that serve as a link to the adolescent’s past and their identity narratives, and this enables them to embrace themselves. Personal belongings, such as photos, stuffed animals, letters and citations, create a closeness to family and friends and becomes a way of expressing and preserving ones sense of belonging and coherence when other trusting moments are absent. The stories tell not only of the significance of influencing the spatial environment through interior design and permission to bring in personal belongings but also of being able to relate and feel a sense of belonging and community.

We’ve slept in each other’s rooms, you don’t get to do that, but I hate to sleep alone, I think it’s really hard. (…) No one likes to be alone here (…) everyone feels bad in their room. (…) Almost everyone here is used to moving around a lot and then it’s like you don’t feel at home and you don’t want to be alone in your room because many are afraid of hurting themselves. (…) Last week we slept three here. (…) You fall asleep so much more beautiful and feel so much better. You don’t feel alone. If there is anything, one can always tell the other. It’s like this with community type. (…) They (the staff) are very disconcerted as soon as they find out that we are sleeping on each other’s rooms because they are afraid that we have beaten each other or such stuff (but) no one who fights would think of staying on the other’s room. You don’t do that.

At a distance, and mostly out of reach, is the desirable and hopeful life waiting to be realized. Thus, the residential youth home can also be a space of distant *hope, longing and desire*—a place where trust and joy are created through meaningful activities, actions and interactions. When the institution presents itself like this, it creates feelings of happiness and belonging. However, most often the institution evokes the opposite emotions, leaving the adolescents longing for a distant future or a past or imaginary space of hope and desire, which is sought but often unattainable or never existed. The adolescents are then left to look after themselves once again.

Some (of the quotes) are about love (…) the ties to those you love, you can be reminded at times. Some, like ‘sometimes you win, sometimes you learn’, are to being able to handle the situation.

It’s from my mother. She has sent two letters here. (I1: And why did you put them on the wall?) My mother sent a letter, it meant a lot. And [buddy] is a big part of me. I want to pay tribute to her for how much she has helped me. Those who are out there do not get in touch so much. It means a lot to the person sitting in there to talk to someone out there.

Hope and dreams are created in the space by attributes, such as personal belongings, that reflect personal memories, longing and belonging. When the institution shows itself to be a trusting and hopeful space, it creates movement—an expansion of both its social and spatial character. To move is to stretch one’s body in physical activity to go beyond the confines of the institution. To move is also to stretch one’s thoughts and allow them to meet other people’s thoughts and worlds. Such movements come to contrast with the passiveness that otherwise characterizes every-day life in the institution.

Sometimes if I have Swedish lesson I read in a book and then I usually go down to her [the art teacher], if she writes on the computer then I can sit next to her and read. It feels nice, I am glad that [the art teacher] is in this school, if she had not been here I probably would not have gone to school at all.

### Comprehensive understanding

The last step in the analysis process comprised the formulation of comprehensive understanding (Lindseth & Norberg, 2004). For this final interpretation, theoretical perspectives on philosophy of existence (Ricœur, 1992) and theoretical perspectives on social exclusion, control and the construction of social problems (Cohen, 1985; Davidsson & Petersson, 2018; Loseke, 2001; Ugelvik, 2014) were used. According to our comprehensive understanding, the socio-spatial environment of the youth institution is perceived as an intrusive and impermeable lived space with occasional moments of light and hope. Time is intertwined with and inseparable from the lived space of the institution, but in contrast to outside life, everything “inside here” is slow and viscous—outside is movement, inside is stasis. The overall experience of the space of the institution has both a spatial, concrete dimension and an abstract, social and existential dimension. These dimensions interplay and appear to strengthen each other, usually causing feelings of loneliness and exclusion. However, the adolescents themselves are trying to catch or create moments of light and hope behind the institutional walls. Sometimes, they appear to get help with this through trusting relationships; however, often they are left behind and try to embrace, nurture and create hope through their own force of will. This emerges as a back and forth process, wherein the adolescents try to get out and get moving, while the every-day socio-spatial life of the institution constantly pulls them back in, encapsulates them and pacifies them. This is a tug of war that appears to be more or less tangible depending on how dense the walls of the institution are—how familiar and invasive the socio-spatial environment that compulsory care entails is to the adolescents inhabiting them. In the socio-spatial context of the institution, negotiation, adaption and consideration therefore become evidence to present oneself as worthy of gaining leave from the controlling, alienating and pacifying institutional spaces and thus gaining access to the intimate, hopeful and desirable spaces outside the walls. To be worthy of access, ultimately to society again, is to enter the road to life, while, not being worthy and denied access is to be left behind.

## Discussion

We sought to understand the children and adolescent’s lived experiences of the socio-spatial environment of the institution. A micro-level perspective on the existential and interactional dimensions of space are necessary to understand and explain the meanings of being incarcerated when focusing on the socio-spatial environment. Meanwhile, the legal, historical and societal context sets the framework for designing the physical and spatial environment, which affects the conditions for institutional practices in everyday life.

Although the interventions at the special youth homes are defined as care and treatment, the lived socio-spatial environment appears to be characterized mainly by security. The adolescents’ stories show a clear awareness of security, incarceration and the power structures existing in these settings. Although strict social control has proven to reduce aggressiveness and violence between inmates (Ulrich et al., 2008), it has at the same time a dehumanizing effect, which, in the worst cases, increases the risk of escapes and suicide (Huey & McNulty, 2005; Svensson, 2010).

Previous studies have shown the need to feel safe and trust caregivers for treatments to have the desired effect (van Der Helm, Stams, van Der Stel, van Langen, & van Der Laan, 2012). Earlier research also showed that the lack of stable relationships and support from caregivers and family, lead to a self-protective form of resilience including avoiding others, not counting on their support (Nourian, Mohammadi Shahbolaghi, Nourozi Tabrizi, Rassouli, & Biglarrian, 2016). However, spatial and temporal conditions also affect the relationships with caregivers, resulting in greater asymmetries during the evening and night (Borge & Fagermoen, 2008). Although the relationship between the person who cares and the person being cared for can never be completely symmetrical, there must be a degree of reciprocity. According to Ricœur (Ricœur, 1992), it is in concrete action that care as an ethic is realized. The care of others and friendship become central to this, as well as a sense of justice within societal institutions (Ricœur, 1992). Without reciprocity and ethical awareness, there is a risk that the relationship will turn into a demonstration of power, whereby one party dominates the other, misusing the caregiver’s expert knowledge and resulting in an asymmetric relationship (Ricœur, 1992) that will undermine the outcomes of care for the children and adolescents. Therefore, the presence of trustful relationships and meaningful social interactions could be seen as fundamental for treatment and for promoting prosocial behaviour and rehabilitation. However, the youth institution appear to be an environment in which intrusion itself position its actors and condition their social interactions (Ugelvik, 2014).

Being incarcerated (a spatial and social exclusion from society) entails a constant awareness of also being excluded from certain spaces at certain times *within* the institution, and, even more, socially distanced from the caregivers. The institutional socio-spatial environment appears to condition actions and interactions and hence the possibility of fruitful treatment alliances, reinforcing the general stigma and exclusion processes that likely existed prior to the institutional placement (Ugelvik, 2014). The institution’s separation practice appears to divide and emerge into further separating practices within the institution (Cohen, 1985).

Previous research has shown that predictability and the ability to control various environmental aspects are required to avoid the experience of deprivation, loss of control and feelings of helplessness (Evans, 2003; Huey & McNulty, 2005; Notley et al., 2012; Svensson, 2010)—experiences that may also increase the risk of suicide (Huey & McNulty, 2005). Moreover, the opportunity to regulate social interaction through access to private areas and the opportunity to choose levels of interaction could influence mental health and create sustainable recovery over time (Evans, 2003; Notley et al., 2012). The reinforcement of social exclusion by socio-spatial control mechanisms, such as rules, regulations and spatial attributes, not only creates feelings of loss of control but also generates a view of the adolescents as (potential) perpetrators, which in turn also risks amplifying the image of the adolescents as problematic in their own eyes (Kitsuse, 1962; Loseke, 2001; Ugelvik, 2014). Besides the pain and sleeping difficulties caused by the environment, the lack of personal control and choice, associated with a body embedded in an alienating environment appears to add to an already destructive self-image and behaviour.

According to Ricœur, self-respect and trust in other people are closely linked through a dialectic movement through stories and relationships with others (Ricœur, 1976). Goffman described how everyday life is performed in the setting of lived and perceived space, where both social and spatial aspects affect the performance and presentation of self and direct it towards the explicitly or implicitly desired behaviour (Goffman, 1990). Similarly, Foucault states that power is dependent on and closely connected with spatial aspects, which affect social roles through invisible borders (Foucault, 1982). Using Goffman’s concept of self-presentation (Goffman, 1982), Enell described how adolescents in assessment situations at special youth homes often felt that they had no control over their self-presentations (Enell, 2015). This will be accentuated by the environmental setting through coercion and the locked units, resulting in the adolescents not recognizing themselves. The adolescents often found that they were required to accept other’s perceptions of them as “troubled children” in a setting where “words had less impact than actions” (Enell, 2016, p. 28) (Enell, 2015). Similarly Holstein (2014), writes about “the system” (an analogy for the various societal institutions) that is everywhere and nowhere, a device that clients in social services meet and try to influence. The system should be seen as an undivided homogeneous whole without a face that is nonetheless represented by individuals who speak for the system (Holstein, 2014). Accordingly, Goffman’s description of the institutionalization processes, where the inmate/patient must adopt the rules and routines of the total institution through different rituals ascribed by the staff, could be seen as another expression of the system (Goffman, 1991). Against this, the socio-spatial environment can be seen as an additional “other”—a feature that the adolescents interact with, are affected by and try to influence. The environment per se not only distances the adolescents and the staff from one another and conditions their actions and interactions but also becomes a third party—an “other” without a face, i.e. an additional representative for the system—whom the adolescents reflect their self on in the absence of others selves. By negotiating with their own behaviour and identity, the adolescents strive to present themselves as worthy of increased degrees of freedom and thereby win access to various spaces within the various layers of the institution. However, the socio-spatial other also appears to reflect the system’s view of the adolescents. With the security and criminal justice logic most often conquering the care and treatment logic, which is reflected in spatial and physical features, words appear to have less impact than the institutional setting itself. The adolescents project their self on the silent socio-spatial other, who answers through the staff with rewards or punishments in time and space: access given or denied. Through the dialectic movement between the self and the socio-spatial other, the adolescent sees and understands themselves as worthy or not worthy (Ricœur, 1976). Being granted access to desirable spaces requires reliability and hence means that the desired worthy character from the perspective of society has been accomplished (Cohen, 1985; Goffman, 1982). Conversely, being denied access implies unreliability and means that one is not (yet) worthy of increased degrees of freedom or, ultimately, exit from the institution.

The result appears as a constant struggle between the system’s practices on the one hand, manifested in spatial attributes and the staff’s actions, and the adolescents’ practices of resistance on the other hand. Acts of resistance, such as scribbling on the walls, smuggling in food or sneaking into each other’s room at night become a kind of counter-ideology to mark distance and disapproval (Kitsuse, 1962; Ugelvik, 2014). However, it also appears that the adolescents attempt to preserve their self and make sense of their life narratives by trying to take control of spatial aspects of their everyday life. In a controlling environment that lacks trustful relationships, this becomes a way to create a safe space for oneself as well as a space to help each other and escape the constant gaze of surveillance and assessment (Enell, 2015; Enell, Gruber, & Andersson Vogel, 2018). More often, however, resistance appears to be interpreted as evidence of problematic behaviour rather than a rational response to a perceived injustice—an act of resistance induced by the very power they are subject to (Foucault, 1982). This appears as a circular process, whereby the system risks reacting with solutions to the (perceived) problem (resistance), and thus once again determining the adolescents as problematic (Emerson & Messinger, 1977; Enell, 2015). The institution’s external spatial boundaries may be clearly visible. However, the boundaries *within* the institution appear to be anything but clear. Giving the adolescents’ capacity to understand and adapt to the socio-spatial environment of the institutional life, this becomes crucial for ultimately winning an exit. However, it appears that the cost is often their self-perception.

## Strengths and limitations

The method chosen for collecting data in the setting of the special youth homes was appropriate; however, some adjustments were required due to specific contextual challenges—for example, security issues and the adolescents’ needs. The method enabled a flexible process and a power shift from interviewer to interviewee. This allowed a responsiveness in the data collection process that is difficult to achieve with other research methods.

Both girls and boys were included in the study. However, the spread of age among the respondents (15–19 years) does not represent the actual spread of age at the special youth homes (11–21 years). The average age of adolescents enrolled at the special youth homes in 2017 was 16 years, with 30% younger than 15 years (The Swedish National Board of Institutional Care (Statens Institutionsstyrelse S), 2017). It is reasonable to assume that both younger children and older adolescents are affected by the socio-spatial environment, with the possibility that this experience is worse for the youngest age groups.

To bond and build trustful relationships with the children/adolescents the researchers spent an extensive time at each unit. This was necessary due to the fact that data collection must be conducted when possible in the view of the youth homes, but also to gain the confidence of the adolescents. There is a fine line between building trust and influencing the respondents to participate in the study, why there is a risk that the results may have been affected by the extensive time spent at site.

The photos facilitated the interview process, since they enabled the adolescents to express their experiences about the abstract phenomenon of the socio-spatial environment, and this generated rich and detailed data. However, the researchers experienced some difficulties as the staff were cautious about what aspects of the environment the adolescents would photograph or the adolescents themselves were not keen to take photos for various reasons. In some cases, no photos were taken for security and ethical reasons. This might have had a negative impact for the target group to verbalize experiences and feelings at the occasion of interviews.

The data analysis method chosen, which was appropriate for the extensive empirical material, put high ethical demands on the researchers. The naïve understanding was subject to minor revisions following dialogue among the research group. The process of analysis was mainly carried out by two of the authors and was discussed at each stage with the other authors, which strengthened the trustworthiness.

The researchers have experience in the present field of research, although none have any personal or work related relation to the institutions and/or the units in the present study. However, there is a potential risk of over interpretation due to preunderstanding. This was handled within the research group, by extensive dialogue prior to data collection, as well as during the analysis process. The first author has work experience of community based and institutional youth care, all other authors have experience from research in regard to the phenomena of interest.

Research in the context of institutional youth care requires thorough discussions regarding ethical standpoints and reflections throughout the research process. From a research perspective, the socio-spatial institutional environment, with its contradicting logics of security and criminal justice versus care and treatment, is important to study, while also what constituting ethical challenges.

To what extent the findings from this study can be generalized to the lived experiences of other institutionalized adolescents cannot be known. Instead, the contribution of this study is to shed light on adolescents’ lived experiences of institutionalization and specifically their experiences of the socio-spatial environment of their everyday institutional lives.

## Conclusions

According to Swedish laws and regulations, all citizens have an equal right to receive high quality care, including the right to integrity, self-determination and partnership (SFS 2014:821, 2014; SFS 2017:30, 2017). Further, all decisions concerning a child or adolescent should be guided by the principle of doing what is in the child’s best interest (SFS 2014:821, 2014; SFS 2017:30, 2017; Swedish Government Offices Social Affairs, 1990; The Social Services Act, 2001; United Nations Human rights office of the high commissioner, 1989), and care environments for vulnerable children should promote the child’s health, self-respect and dignity (United Nations Human rights office of the high commissioner, 1989). A socio-spatial care environment that meets the requirements of law must therefore start from the child’s best interests with the intention of strengthening integrity, self-respect and dignity. However, the Ombudsman for Children in Sweden, and the United Nation Committee on the Rights of the Child have strongly criticized special youth homes in general and their specific powers in particular (Children’s Ombudsman, 2019; Sandberg Lööf, 2011; United Nations Committee on the Rights of the Child, 2015).

By employing a micro-level perspective on the existential and interactional dimensions of lived space, the analysis shows that adolescents’ experience of the environment is characterized by a constant struggle to appear worthy of increased degrees of freedom in space and time. Their negotiation with their behaviour may give increased degrees of freedom but may incur a cost regarding their self-perception. When applying a macro-level perspective to understand the youth institution in a wider social context, the external spatial boundaries of the institution appear clear; however, the boundaries inside the institutional socio-spatial environment appear to be blurred and associated with rewards and punishments. Although the special youth homes are said to perform according to a care and treatment profile, it appears that a security and criminal justice logic guides the spatial design and socio-spatial environment. To put a child or adolescent under involuntary treatment through the application of the law requires legal intervention and hence places high demands on the state. However, the present socio-spatial care environment of the special youth homes appears to leave out crucial ethical and child rights perspectives in favour of security issues and reprisals. The dichotomy of care and treatment ideals on the one hand and security ideals on the other has been highlighted in the text, but it needs to be more thoroughly investigated in future research, both in terms of quantitative measurements and further qualitative perspectives, such as the staff’s point of view.
